# An Ensemble Outlier Detection Method Based on Information Entropy-Weighted Subspaces for High-Dimensional Data

**DOI:** 10.3390/e25081185

**Published:** 2023-08-09

**Authors:** Zihao Li, Liumei Zhang

**Affiliations:** School of Computing, Xi’an Shiyou University, Xi’an 710065, China

**Keywords:** high-dimensional data, outlier detection, information entropy, subspaces, ensemble

## Abstract

Outlier detection is an important task in the field of data mining and a highly active area of research in machine learning. In industrial automation, datasets are often high-dimensional, meaning an effort to study all dimensions directly leads to data sparsity, thus causing outliers to be masked by noise effects in high-dimensional spaces. The “curse of dimensionality” phenomenon renders many conventional outlier detection methods ineffective. This paper proposes a new outlier detection algorithm called EOEH (Ensemble Outlier Detection Method Based on Information Entropy-Weighted Subspaces for High-Dimensional Data). First, random secondary subsampling is performed on the data, and detectors are run on various small-scale sub-samples to provide diverse detection results. Results are then aggregated to reduce the global variance and enhance the robustness of the algorithm. Subsequently, information entropy is utilized to construct a dimension-space weighting method that can discern the influential factors within different dimensional spaces. This method generates weighted subspaces and dimensions for data objects, reducing the impact of noise created by high-dimensional data and improving high-dimensional data detection performance. Finally, this study offers a design for a new high-precision local outlier factor (HPLOF) detector that amplifies the differentiation between normal and outlier data, thereby improving the detection performance of the algorithm. The feasibility of this algorithm is validated through experiments that used both simulated and UCI datasets. In comparison to popular outlier detection algorithms, our algorithm demonstrates a superior detection performance and runtime efficiency. Compared with the current popular, common algorithms, the EOEH algorithm improves the detection performance by 6% on average. In terms of running time for high-dimensional data, EOEH is 20% faster than the current popular algorithms.

## 1. Introduction

Outlier detection refers to the process of identifying abnormal data points within a normal dataset. Outliers are characterized by their deviation from normal data and often constitute a small proportion of the total dataset [1]. The prevalence of outliers and their influence on the total dataset means that outlier detection is an important task in the field of data mining and a highly active area of research in machine learning. Researchers from various disciplines, including statistics, big data, and machine learning, have shown a keen interest in outlier detection. Additionally, outlier detection plays a significant role in various applied domains, such as network intrusion detection in computer systems [2,3,4], fraud detection in credit card transactions [5,6], and anomaly detection in health insurance [7,8], to name just a few.

Many datasets based on the real world, in order to fully describe the reality, are often high-dimensional, where the number of dimensions can reach hundreds of thousands. With the increasing number of data dimensions faced by anomaly detection, many conventional anomaly detection algorithms have lost their function, and the “dimensional curse” [9] has brought some difficulties to anomaly detection. One issue is that high-dimensional data become sparse during full-dimensional analyses. Aggarwal’s 2013 study demonstrated that in sufficiently high-dimensional spaces, data points tend to exhibit an almost equidistant distribution, known as the sparsity of high-dimensional data [10]. As the definition of anomalies is directly related to the position of data points distributed in sparse regions, this has a catastrophic impact on anomaly detection.

**Figure 1:**
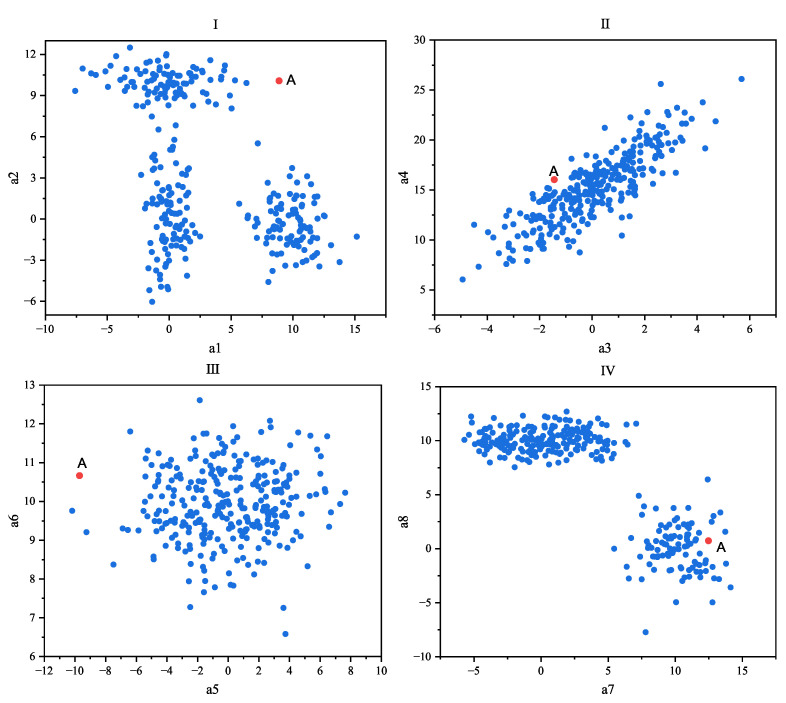
Distribution of anomalous data point A in different subspaces.

Another difficulty is that an object may possess hundreds or even thousands of measured and observed attribute features, but its anomalous behavior may only manifest in a small subset of these features [11]. By focusing on a subset of attribute feature values or subspaces, it is possible to discover anomalous data. However, when data are represented in full dimensionality, anomalies reflected in only a few attribute features can be obscured by the variation and noise present in the massive amount of otherwise normal data. It should be noted that noise is a random error or bias in an observed quantity, including wrong values, that deviate from the desired point. Noise will not only increase the amount of data, but will also increase the amount of calculation required to process the data; it will increase the memory and calculation overhead, and also increase the calculation error. Outliers are due to systematic errors, human factors, or inherent data variations that make some data inconsistent with the behavior of the population. The noise points are often irregular and weak, so it is not necessary to analyze them. Outliers usually have a higher degree of noise while being more explanatory. Figure 1 illustrates a dataset with eight dimensions (a1, a2, a3, a4, a5, a6, a7, and a8), displayed in different subspaces that were formed by pairwise combinations of dimensions. It can be observed that data point A was identified as an anomaly in Subspace I and Subspace III (a1a2 and a5a6), while it was detected as a normal data point in Subspace II and Subspace IV (a3a4 and a7a8). Thus, from the perspective of measuring the anomaly value of point A, these subspaces contain noise. In this scenario, two out of the four subspaces provide little or no information for detecting whether point A is anomalous and is subject to noise. Consequently, when performing distance-based anomaly detection with full dimensionality, anomalies may be lost within these subspaces. This issue is naturally exacerbated as the number of dimensions increases. For high-dimensional datasets, only a very small subset of subspaces may be genuinely helpful in the identification of outlier points. For example, in a car-fault detection scenario, the results of thousands of different vehicle tests (horn, brakes, axle load, chassis, ignition system, lighting system, exhaust system, etc.) performed on the same car may be mostly normal with some noise variations that are not significant. On the other hand, some deviations present in a small fraction of these tests may be significant enough to indicate anomalous behavior. When the data from the test are represented in full dimensions, anomalous data points will not appear significant in almost all except for a very small subset of the dimensions of the data. Therefore, for high-dimensional data, conventional detection algorithms are less likely to expose outliers as they are masked by the noisy variations of a large number of normal tests [12]. Therefore, through the idea of statistics and/or machine learning, selecting specific dimensions that can reflect significant anomalies such that they form a subspace, as well as performing anomaly detection in the subspace, can greatly improve the accuracy and efficiency of the algorithm.

Effectively identifying anomalies from within high-dimensional data is a challenging problem in anomaly detection. To pursue this topic, numerous researchers have proposed a variety of detection algorithms in order to address these challenges. Generally, these algorithms can be categorized into three types of methods: neighborhood-based (e.g., RBDA) [13], subspace-based (e.g., SOD) [14], and ensemble-based (e.g., HiCS) [15].

### 1.1. Neighborhood-Based Detection

The basic idea behind neighborhood-based anomaly detection methods is to utilize neighborhood information to identify anomalous values. Given a data point, the anomaly score is defined as the average distance to its k-th nearest neighbor (KNN) or weighted distance (KNNW). Wang et al. [16] proposed an adaptive KNN algorithm based on Support Vector Data Description (SVDD) to determine the validation set and introduced a dynamic classifier selection into the anomaly detection. Another strategy is to use the Local Outlier Factor (LOF) to measure to what degree data are anomalous and where the anomaly score is relative to its neighborhood measurement. The LOF-based method provides a score that reflects the anomaly level for each data point. The resulting score is interpretable and can be compared within a dataset. To address the issue of poor detection performance and the low robustness of local methods for datasets with complex distributions, Renmin et al. [17] proposed a new local dynamic neighborhood-based detection algorithm (LDNOD). They designed a novel nearest neighbor computation method called Dynamic Reference Nearest Neighbor (DRNN), which is based on dynamic references rather than simply a monotonic search for neighboring data points.

All the aforementioned neighborhood-based detection methods are capable of detecting outlier objects under certain conditions. However, their performance heavily relies on distance measurements such as Euclidean distance, which become unstable or even meaningless in high-dimensional spaces.

### 1.2. Subspace-Based Detection

Anomalies are more likely to exhibit anomalous behavior in multiple local or low-dimensional subspaces. This type of low-dimensional or local anomalous behavior can often be overlooked when conducting an overall high-dimensional analysis. Zimek et al. [18] pointed out that, for objects in high-dimensional spaces, only a subset of relevant features actually provide meaningful information for anomaly detection, while the remaining features are irrelevant for detection. The presence of irrelevant features hinders the performance of anomaly detection models. Subspace learning is a popular technique in the relevant literature for dealing with high-dimensional spaces, and it has been the subject of extensive research in anomaly detection. Subspace-based anomaly detection methods aim to discover anomalous behavior by selectively screening different dimensional subspaces in an ordered manner. These methods come in two representation forms: sparse subspace methods [19,20] and correlated subspace methods [21,22]. Dutta [23] proposed a sparse coding framework-based outlier detection algorithm called the Rare Outlier Detection via Sparse coding (RODS) algorithm, which consists of two parts: NLAR learning and an outlier detector. RODS offers a new definition for outlier detection that is useful for high-dimensional data. SOD [18] is a typical example of a correlated subspace learning method. It first explores multiple correlated datasets by using the nearest neighbors of each object, and then determines the axis-parallel subspaces on each correlated dataset based on their linear correlation in order to reduce the variance of each feature in the subspace. Finally, anomaly detection is then performed based on the correlated subspaces.

In addition to being computationally expensive, the primary limitation of this detection method is that it requires a large amount of local data to detect bias trends and to determine the selection of subspaces.

### 1.3. Ensemble-Based Detection

Ensemble learning has been widely studied in machine learning [24], and due to its superior performance compared to other related techniques, it is also frequently used in anomaly detection. Given the characteristics of high-dimensional data, no single basic anomaly detection method can effectively discover all of the anomalies in high-dimensional space. Therefore, researchers have started to explore ensemble learning to address the detection of anomalies in high-dimensional space [25]. One common ensemble method is bagging (bootstrap aggregating), where multiple different subspaces or subsamples are used with either the same or different base detectors. The anomalies are then determined through the ensemble aggregation of anomaly scores or rankings. In anomaly detection, bagging methods can be divided into two categories: feature-based bagging and sample-based bagging. One classic study providing an example of feature bagging detection is “Feature Bagging for Outlier Detection” authored by Lazarevic and Kumar in 2005 [26], wherein feature subsets are randomly selected from the original feature space. On each feature subset, an outlier detection algorithm is used to estimate the anomaly score for each object. Subsequently, each of the scores for the same object are integrated into a final score. In this study, each base detection algorithm is based on the LOF algorithm. Nguyen et al. [27] expanded upon this approach by using different base detection algorithms for different subspaces to estimate the anomaly scores of objects in random subspaces. Using an adaptation of the feature bagging (FB) approach, Wang et al. [28] proposed a selective feature bagging (SFB) detector that simultaneously considers variance and bias reduction. To improve accuracy without compromising the diversity of FB base detectors, these researchers employed the concept of dynamic classifier selection. Unlike Nguyen’s approach, Wang’s method dynamically selects the most effective base detector for each subspace. The earliest study on using data bagging detection for anomaly detection algorithms was conducted by Zimek [29], who expanded the diversity of the testing samples for detecting anomalies. There are also several anomaly detection methods that consider both feature bagging and data sub-sampling. For example, J.R. et al. [30] obtained different features through feature bagging in each iteration and calculated anomaly scores for different data subsets by using sub-sampled sets.

Although the above two ensemble-based detection methods have achieved relatively successful results in addressing high-dimensional space problems, they rely on the randomness of samples to improve the diversity of detection results, resulting in a large amount of redundant computation that results in a waste of time and space.

In addressing the aforementioned issues, this paper makes the following contribution. Proposing a novel anomaly detection algorithm called EOEH.

1. By employing a subsampling technique based on ensemble thinking, this study introduces diversity into the ensemble of density-based unsupervised anomaly detection methods. In each subsample, the densities around each data point in the dataset are determined to compute their respective anomaly scores. This approach provides diversified detection results, reduces global variance through ensemble learning, and enhances the robustness of the algorithm model.
2. To tackle the redundancy and irrelevance of the numerous subspaces present in high-dimensional data, this paper proposes a weighted subspace outlier detection algorithm based on information entropy. This algorithm determines the corresponding weighted subspaces based on the information entropy of data points in each dimension. It redefines the distances between data points, thereby reducing the noise impact caused by high-dimensional data and improving density-based outlier detection performance for high-dimensional data.
3. This study optimizes algorithm implementation based on the LOF (Local Outlier Factor) algorithm framework, enhancing the differentiation between normal and anomalous data, as well as making anomalous behavior more salient. This improvement results in an overall enhancement of the algorithm model’s anomaly detection performance.

The rest of the paper is organized as follows. Section 2 presents a review of related work. Section 3 introduces the EOEH algorithm model. Section 4 reports the EOEH algorithm model’s experimental results. Finally, in Section 5, a conclusion is offered based on the algorithm’s performance.

## 2. Materials and Methods

Generally, anomaly detection algorithms operate in one of the following three ways: supervised anomaly detection, unsupervised anomaly detection, and semi-supervised anomaly detection. The main characteristic of supervised anomaly detection is that the dataset is labeled. Unsupervised anomaly detection, on the other hand, operates in a learning environment where the data are unlabeled. Semi-supervised anomaly detection utilizes both labeled and unlabeled data. The Local Outlier Factor (LOF) algorithm is a classic example of unsupervised anomaly detection [31,32].

At the same time, the LOF technique is also a density-based outlier detection algorithm, which can be used to solve the detection difficulties caused by data skew or those incurred due to no clear statistical characteristics across the data regions. Therefore, traditional distance- and domain-based outlier detection methods do not work because they assume that all input data obey the same distribution, and due to them then using global criteria to judge whether the data are outliers. In addition, traditional distance- and domain-based outlier detection methods cannot make full use of the neighborhood information of the data. Instead, the LOF method exploits the relative density of each point and its k-nearest neighbors to detect outliers. Since the local density can better reflect the true distribution characteristics of each data, the LOF algorithm has a better detection effect on skewed data. Since most data streams in the real world are skewed data, the LOF algorithm outperforms other methods in a wide range of applications [31].

Researchers from various fields have made improvements and extensions to the LOF algorithm based on the requirements of specific applications. Tang et al., introduced the Connectivity-based Outlier Factor (COF) algorithm [33], which improves the way in which density estimation is performed. Papadimitriou et al., addressed complications that surround choosing the k value and proposed the Local Correlation Integral (LOCI) algorithm [34]. The LOCI algorithm considers all possible k values for each data point and selects the point with the highest score to serve as the k value. The Local Outlier Probability (LOOP) algorithm [35], proposed by Kriegel et al., in 2009, assumes that the distances from the data points to their nearest neighbors follow a Gaussian distribution. To address the limitations of existing methods that focus only on the overall separation between objects and their neighboring objects while ignoring their dispersion, Su et al., proposed the Efficiently Exploring Dispersed Local Outliers using Density-based Local Outlier Score (E2DLOS) algorithm [36]. This algorithm redefines the Local Outlier Factor as the Local Deviation Coefficient (LDC) by leveraging the distribution of objects and their neighbors. Building upon the LOF algorithm as a base detector, Mahboobeh et al. [37] proposed the MRD and mRMRD methods based on the use of mutual information for subspace selection. These methods have made significant progress in addressing the limited effectiveness of density-based anomaly detection in high-dimensional data. In the proposed algorithm model EOEH, the LOF algorithm is improved and optimized again. Compared with the algorithms of other researchers, this paper puts forward higher requirements for detection results, reconsiders the definition of reachable distances in the LOF method, further subdivides the local relative positions of each data, expands the differentiation between normal points and outliers, and improves the detection performance.

This paper is not simply on improving and optimizing the LOF algorithm, but also about studying integration-based detection ideas and subspace-based detection ideas (combined with sub-sampling technology), and using information entropy to construct a new index weight calculation method, thus proposing a new index weight calculation method. The brand-new algorithm model EOEH was created to solve the detection challenge in high-dimensional space. The basic detector is to redefine the reachable distance, the local reachable density and local outlier factor of the LOF algorithm, as well as to obtain a high-precision field-based and density-based detection algorithm HPLOF.

In the following, the modifications and innovations of the EOEH algorithm model on the original algorithm are briefly introduced. In this model, a random subsampling of data is first performed, which involves running the detectors on different small-scale sub-samples. This approach provides diverse detection results, and by integrating these results, the algorithm’s detection performance is improved.

Next, an information entropy-based method is employed to calculate the weights of the dimensions in the feature space. This method helps to differentiate between the various influence factors in different dimension spaces, allowing for the generation of anomaly subspaces and weights for data objects in response to these dimensions. This helps to reduce the noise impact of high-dimensional data, as well aids with improving anomaly detection performance for these data.

Finally, a new High-Precision Local Outlier Factor (HPLOF) detector is designed. This detector enhances the differentiation between normal and anomalous data, thereby improving the robustness of the algorithm model.

**Figure 2:**
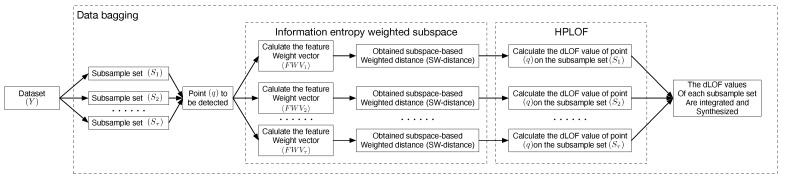
Conceptual diagram of the EOEH algorithm model.

The conceptual diagram of the algorithm model is shown in Figure 2.

## 3. EOEH

### 3.1. Subsampling

Subsampling is a technique based on the idea of ensembles, and it involves randomly sampling the original dataset to create multiple distinct sub-samples. This process helps to introduce diversity into unsupervised anomaly detection methods and reduces the overall variance. In each sub-sample, the density around each data object is determined to calculate each object’s overall anomaly score.

It is important to note that performing simple subsampling and running an anomaly detection algorithm on these sub-samples may fail to detect anomaly information for many objects. Some anomalous objects may not be included in any of the sub-samples, and many objects may only obtain correct anomaly scores from certain sub-samples but not others. On the contrary, for each subset, a sample is extracted from the dataset, and the neighborhoods of each object in the dataset are calculated based on the subset. Therefore, using a sub-sample-based ensemble can result in significant speed improvements compared to other types of ensembles. Even for sub-samples and ensembles that are small in size, this method can deliver results comparable to running the algorithm on the entire dataset.

### 3.2. Information Entropy-Weighted Subspace

Entropy is an important tool in information theory that is used to describe the uncertainty of information and random variables [38]. Let X be a random variable with a set of possible values S(X), and let P(x) represent the probabilities of X taking on different values. The entropy of X is defined as shown in Equation (1).

(1) $$E\left(X\right)=-\sum_{x\in S(X)}P\left(x\right)\cdot \log_{2}\left(P\left(x\right)\right)$$

Based on the principle that greater uncertainty in a variable corresponds to higher entropy [39], more information is required to increase the clarity and visibility of a variable. Conversely, lower entropy indicates less uncertainty.

Christian et al. proposed an idea called local subspace preference in the literature [40], which assumes that the attribute set of a d dimensional dataset *D* is $A=\left\{A_{1},\cdots ,A_{d}\right\}$, and that the projection of a data point p in D onto an attribute $A_{i}$ is denoted as $\Pi_{A_{i}}\left(p\right)$, where $N_{\varepsilon }\left(p\right)$ is the ε neighborhood of *p* (ε is the distance radius). The relevant definition is as follows:

**Definition** **1.** *Variance along an attribute, where the variance of $p\in D,\;A_{i}\in A$, N${}_{\varepsilon }\left(p\right)$ with respect to $A_{i}$ is defined as*

(2) $$VAR_{A_{i}}\left(N_{\varepsilon }\left(p\right)\right)=\frac{\sum_{q\in N_{\varepsilon }\left(p\right)}{\left(\mathrm{dist}\left(\Pi_{A_{i}}\left(p\right),\Pi_{A_{i}}\left(q\right)\right)\right)}^{2}}{\left|N_{\varepsilon }\left(p\right)\right|}$$

**Definition** **2.** *Subspace preference dimensionality for p∈D, δ>0, if $VAR_{A_{i}}\left(N_{\varepsilon }\left(p\right)\right)⩽\delta$, call $A_{i}$ the subspace preference dimensionality of $N_{\varepsilon }\left(p\right)$.*

Based on the idea of local subspace preference, we propose an entropy-based abnormal feature subspace selection method for outlier detection in high-dimensional data.

Consider a dataset Y with n dimensions and m samples. Its feature set is denoted as $\left\{a=a_{1},a_{2},\ldots ,a_{n}\right\}$. The projection of data point *q* in Y onto an attribute feature $a_{i}$ is represented as $\Pi_{a_{i}}\left(q\right)$ and $S_{t}$ represents a subsample set obtained through the secondary sampling process from the dataset Y. The relevant definitions are as follows:

**Definition** **3.** *The sub-sample feature entropy of data point q with respect to attribute feature $a_{i}$:*

(3) $${\mathrm{SFE}}_{a_{i}}\left(q\right)=-\sum_{p\in S_{t}}\frac{\mathrm{dist}\left(\Pi_{a_{i}}\left(q\right),\Pi_{a_{i}}\left(p\right)\right)-d_{\min}}{d_{\max}-d_{\min}}\cdot \log_{2}\left(\frac{\mathrm{dist}\left(\Pi_{a_{i}}\left(q\right),\Pi_{a_{i}}\left(p\right)\right)-d_{\min}}{d_{\max}-d_{\min}}\right)$$

(4) $$\left.d_{\max\;}=\max\left\{\mathrm{dist}\left(\Pi_{a_{i}}\left(q\right),\Pi_{a_{i}}\left(p\right)\right)|p\in S_{t}\right)\right\}$$

(5) $$\left.d_{\min}=\min\left\{\mathrm{dist}\left(\Pi_{a_{i}}\left(q\right),\Pi_{a_{i}}\left(p\right)\right)|p\in S_{t}\right)\right\}$$

The sub-sample feature entropy $SFE_{a_{i}}\left(q\right)$ describes the distribution of the projection values of other data points in the sub-sample with respect to the attribute feature $a_{i}$ when considering data point q as the reference center. A higher value of $SFE_{a_{i}}\left(q\right)$ indicates greater instability or variability in the behavior of data points with respect to the attribute feature $a_{i}$ around *q*. Conversely, a lower value suggests a more regular or standardized distribution of data points around q on the attribute feature $a_{i}$. After generating the sub-sample feature entropy $SFE_{a_{i}}\left(q\right)$ for data point q across different dimensions, the exceptional subspaces for q can be further defined.

From an information entropy perspective, this increased uncertainty in the sub-sample dataset is caused by the presence of anomalies. The uncertainty exhibited by anomalous points is due to the distribution of their attribute values in certain features. Therefore, by calculating the sub-sample feature entropy of each point on each feature, it is used as the basis for judging whether the feature is an abnormal feature of the point, thereby constructing the abnormal feature subspace. Through its construction, the weight of a point on different features can be obtained, and the distance in the high-dimensional space can be redefined, which finally makes the abnormal behavior in the dataset more obvious.

**Definition** **4.** *If an attribute feature $a_{i}$ satisfies the following conditions, it is referred to as an exceptional feature of data point q:*

(6) $${\mathrm{SFE}}_{a_{i}}\left(q\right)>\frac{\sum_{p_{\in S_{t}}}{\mathrm{SFE}}_{a_{i}}\left(p\right)}{\left|S_{t}\right|}$$

According to Definition 7, if the sub-sample feature entropy of q with respect to $a_{i}$ is more than the average feature entropy of other data points in the sub-sample set with respect to $a_{i}$, it indicates that q exhibits greater uncertainty than other data points in the sub-sample set with respect to attribute feature $a_{i}$. Therefore, the features that satisfy this condition are referred to as exceptional features with respect to q.

**Definition** **5.** *Abnormal Feature Subspace of q (AFS(q)). The abnormal feature subspace of point q is $AFS\left(q\right)=\{a_{i}\in a|$, wherein $a_{i}$ is an abnormal feature of q}. The AFS(q) consists of all the abnormal features of q. This feature subspace corresponds to the projection subspace of the n-dimensional feature space of the dataset, where data point q exhibits high levels of uncertainty.*

### 3.3. HPLOF

To better differentiate between normal and outlier data, as well as to improve the robustness of the algorithm model, this paper proposes a new outlier detector called the High-Precision Local Outlier Factor (HPLOF) algorithm. The HPLOF algorithm is built upon the framework of the original LOF algorithm, but it redefines reachability distance, local reachability density, and the local outlier factor. By introducing a binary division in the reachability distance (tworeach-$dist_{k}\left(q,p\right)$), this algorithm achieves a finer distinction between the distance relationship of a data object and its k-nearest neighbors. This leads to a more detailed local reachability density ($dlrd_{k}\left(q\right)$), which can then be compared to the average detailed local reachability densities of other points in its neighborhood. Ultimately, this results in a detailed local outlier factor ($dLOF_{k}\left(q\right)$) that allows for the enhanced capture of anomaly behavior.

The HPLOF algorithm optimizes and improves the LOF algorithm based on the weighted distance of information entropy, which is a new concept of measuring the local information of a point in the LOF algorithm (namely the two-step reachability distance) that is defined. This further expands the difference between the abnormal value and the normal value, makes the abnormal behavior more significant in the score, improves the detection accuracy, and endows the LOF algorithm with higher accuracy.

**Definition** **6.** *Two-step reachability distance is based on k. Let q be an arbitrary data point in the dataset, and let p be a point in its k-neighborhood (specifically the ra-th closest point to q). The two-step reachability distance based on k between data point q and data point p is defined as follows:*

(7) $$two\;reach-dist_{k}\left(q,p\right)=\left\{\begin{array}{c} \left\{\begin{array}{c} ra-diatance(p),d(q,p)\leq ra-distance(p)\\ k-diatance(p),d(q,p)>ra-distance(p) \end{array}\right.,\;q\in \left|N_{k}\left(p\right)\right|\\ d\left(q,p\right),q\notin |N_{k}\left(p\right)| \end{array}\right.$$

The two-step reachability distance is defined based on the k between data points p and q, which is denoted as two $reach-dist_{k}\left(q,p\right)$, where $N_{k}\left(p\right)$ represents the set of k nearest points to data point p,d(q,p), which represents the Euclidean distance between points p and q. Furthermore, k-distance (p) represents the k-distance of data point p, and ra-distance (p) represents the distance between data point p and its ra-th nearest neighbor.

**Definition** **7.** *Detailed local reachability density (dlrd). The detailed local reachability density of a data point q is defined as the reciprocal of the average of the two-step reachability distances based on k between q and its k nearest neighbors. It represents the local density of q relative to its neighborhood. The detailed local reachability density is denoted as dlrd.*

(8) $${\mathrm{dlrd}}_{k}\left(q\right)=\frac{\left|N_{k}\left(q\right)\right|}{\sum_{p\in N_{k}\left(q\right)}\;\mathrm{two}\;\mathrm{reach}\text{-}\mathrm{dist}\;\left(q,p\right)}$$

**Definition** **8.** *Detailed local outlier factor (dLOF). The detailed local outlier factor builds upon the original local outlier factor (LOF) by further refining the outlier degree assessment for the relevant data points. The dLOF enhances the differentiation between outliers and normal points, thereby improving the accuracy and robustness of anomaly detection from a statistical perspective.*

(9) $${\mathrm{dLOF}}_{k}\left(q\right)=\frac{\sum_{p\in N_{k}\left(q\right)}\frac{{\mathrm{dlrd}}_{k}\left(p\right)}{{\mathrm{dlrd}}_{k}\left(q\right)}}{\left|N_{k}\left(q\right)\right|}$$

Similar to the original local outlier factor algorithm, dLOF still represents a density comparison, indicating the difference in density between data point q and its local neighborhood. Therefore, if the dLOF value is larger, it indicates that the data object is more anomalous. Conversely, if the dLOF value is smaller, it suggests that the data object has a density similar to its local neighbors and is therefore more likely to be normal.

### 3.4. Algorithm Implementation

The EOEH algorithm model is divided into three stages: In the first stage, random secondary sub-sampling is implemented, and one section of the original dataset is selected as a sub-sample set without replacement. Using this process, multiple random sub-sample sets are then obtained through repeated selection. In the second stage, based on information entropy, the abnormal features of each data object in the original dataset present in each subsample set are calculated and combined into the abnormal feature subspace corresponding to each data object. Furthermore, the various information entropy weights are set according to whether the dimension belongs to the abnormal feature subspace. In the third stage, each data object in the dataset is calculated on each subsample set, the obtained information entropy weight is used to redefine the distance metric, and it is then imported it into the high-precision local anomaly factor detector to obtain the dLOF value. Finally, the dLOF value obtained for each subsample set is integrated and ranked to obtain the final outlier value.

The following section will provide further explanations on how to utilize abnormal feature subspaces to obtain information entropy weights and how to redefine distance within the algorithm model.

**Definition** **9.** *Feature weight vector. For a data point q ∈ D, the feature weight vector $FWV\left(q\right)=\left\{\omega_{1},\omega_{2},\ldots ,\omega_{n}\right\}$, where each $\omega_{i}$ represents the information entropy weight of feature $a_{i}$ with respect to data point q.*

(10) $$\omega_{i}=\left\{\begin{array}{c} \alpha ,\alpha >\beta ,a_{i}\in \mathrm{AFS}\left(q\right)\\ \beta ,a_{i}\notin \mathrm{AFS}\left(q\right) \end{array}\right.$$

**Definition** **10.** *Subspace-based weighted distance. For q, p *∈* D, where $\omega_{i}$ represents the information entropy weight of feature $a_{i}$ with respect to q. The subspace-based weighted distance between q and p is based on the q’s feature subspace, and is defined as follows:*

(11) $$SW-\mathrm{distance}\left(q,p\right)=\sqrt{\sum_{n}\omega_{i}{\left(\Pi_{a_{i}}\left(q\right)-\Pi_{a_{i}}\left(p\right)\right)}^{2}}$$

The implementation of EOEH algorithm is shown as Algorithm 1.

**Algorithm 1** EOEH**Input:** dataset *Y* with dimension *n* and sample number *m*, number of subsample sets *T*, number of samples in each subsample set μ, abnormal entropy weight α, normal entropy weight β, neighborhood parameter *K* **Output:** Integrate exception score set *O*
1:**Begin**2:**for** $S_{t}\leftarrow 1$ **to** *T* **do**3:    $S_{t}$ = Random(Y,μ)   // The subsample set $S_{t}$ is formed from the dataset *Y* without putting back μ of random sampling4:**End for**5:q∈Y, $p\in S_{t}$, *a* = Feature set *a* = $a_{1},a_{2},\ldots ,a_{n}$ of dataset *Y*6:**for** $S_{t}\leftarrow 1$ **to** *T* **do**7:    **for** $a_{i}\leftarrow 1$ **to** *n* **do**8:        $SFE_{\left(S_{t},a_{i}\right)}\left(q\right)$   // Calculate the subsample eigenentropy of point *q* on the subsample set $S_{t}$ with respect to feature $a_{i}$9:        **if** the $SFE_{\left(S_{t},a_{i}\right)}\left(q\right)>$ the average of $SFE_{\left(S_{t},a_{i}\right)}\left(p\right)$ for other points in the sub-sample set **then**10:            $AFS_{\left(S_{t}\right)}\left(q\right)$ = $\{a_{i}\in a|a_{i}$ is an abnormal feature of q} // Point *q* belongs to the abnormal feature subspace in $S_{t}$, which is formed by all the abnormal features of *q* in $S_{t}$.11:        **End if**12:    **End for**13:**End for**14:The feature weight vector of point *q* in the sub-sample set $S_{t}$ is $FWV\left(q\right)=\left\{\omega_{1},\omega_{2},...,\omega_{n}\right\}$.15:**for** $\omega_{i}\leftarrow 1$ **to** *n* **do**16:    **if** $a_{i}\in AFS_{\left(S_{t}\right)}\left(q\right)$ **then**   //The feature weight vector of point *q* is assigned differently for each feature based on the abnormal feature subspace.17:        $\omega_{i}=\alpha$18:    **else**19:        $\omega_{i}=\beta$20:    **End if**21:**End for**22:**for** $S_{t}\leftarrow 1$ **to** *T* **do**23:    **for** each $p\in S_{t}$ **do**24:        $SW-distance_{\left(S_{t}\right)}\left(q,p\right)$   // Calculate the weighted distance based on the subspace between point *q* and each point in the sub-sample set.25:    **End for**26:    Perform the KNN (k-nearest neighbors) algorithm on sub-sample set $S_{t}$ using the $SW-distance_{\left(S_{t}\right)}\left(q,p\right)$ (weighted distance) metric between point *q* and point *p*.   // Obtain the k-neighborhood of point *q* on the sub-sample set based on the weighted distance.27:    **for** j←1 **to** *K* **do**28:        two reach-$dist_{\left(S_{t},k\right)}\left(q,p\right)$   // Calculate the two-reach distance of point *q* within its k-neighborhood29:    **End for**30:    Based on the definition of detailed local reachability density, it can calculate the $dlrd_{\left(S_{t},k\right)}\left(q\right)$ value of point *q* on the subset $S_{t}$.31:    By averaging the $dlrd_{\left(S_{t},k\right)}\left(q\right)$ value of point *q* and the $dlrd_{\left(S_{t},k\right)}\left(q\right)$ values of other points in its k-neighborhood, it can obtain the detailed local outlier factor $dLOF_{\left(S_{t},k\right)}\left(q\right)$ that reflects the abnormality of point *q* on the subset $S_{t}$.32:**End for**33:By utilizing the ensemble anomaly score $O_{q}=\sum_{T}dLOF_{\left(S_{t},k\right)}\left(q\right)/T$ based on the subset, calculating the ensemble anomaly scores for each data object in the dataset *Y*. The ensemble anomaly score set $O=O_{1},O_{2},...,O_{m}$ is obtained, where $O_{i}$ represents the ensemble anomaly score for the i−th data object in the dataset *Y*.34:**End**

By sorting the ensemble anomaly score set $O=\{O_{1},O_{2},\ldots ,O_{m}\}$ and selecting the data objects with higher ranking scores, the EOEH algorithm is able to determine which data points are abnormal data.

## 4. Algorithm Experiment

### 4.1. Experimental Environment

The experiments included in this study were conducted on a machine with an Intel(R) Core(TM) i5-9300H CPU @ 2.40GHz processor and 8.00 GB of memory. The manufacturer of this machine is Hewlett-Packard Company from Palo Alto, CA, USA, purchased this machine from the official website of HP Company.

### 4.2. Experimental Metrics

In order to compare the detection performance of the EOEH algorithm with other anomaly detection algorithms, this study used the Balanced F Score (F1 score), the Area Under the ROC Curve (AUC), and runtime as the performance metrics through which to evaluate the proposed outlier detection algorithm.

Regarding the ROC curve, in order to understand its implementation, it is necessary to introduce several related concepts. As shown in Table 1, a confusion matrix was used to describe the classification results of a classification problem.

According to the confusion matrix, the recall rate was defined as Equation (12), which represents the proportion of samples predicted by the model to be class 1 among the samples with the true class label of 1. The definition precision is shown in Equation (13), which represents the proportion of samples predicted by the model to be class 1, and the true class label is 1. The F1 score was defined as the harmonic average of the recall rate and accuracy rate, as shown in Equation (14). The F1 score ranges from 0 to 1, with the closer the value is to 1, the better the model’s prediction.

Based on the confusion matrix, the true positive rate (TPR) was defined in Equation (15), which represents the proportion of samples with true class labels equal to 1 that are correctly predicted as class 1. The false positive rate (FPR) is defined in Equation (16), which represents the proportion of samples with true class labels equal to 0 that are incorrectly predicted as class 1. The FPR is plotted on the x-axis of the ROC curve, and the TPR is plotted on the y-axis. The area under the ROC curve (AUC) is a measure of the performance of a classification model and represents the probability that a randomly chosen positive instance will be ranked higher than a randomly chosen negative instance. Simply put, the higher the AUC value, the better the detection performance of the classifier.

(12) $$\;\mathrm{recall}\;=\frac{TP}{TP+FN}$$

(13) $$\;\mathrm{precision}\;=\frac{TP}{TP+FP}$$

(14) $$F1=2\cdot \frac{\;\mathrm{precision}\;\cdot \;\mathrm{recall}\;}{\;\mathrm{precision}\;+\;\mathrm{recall}\;}$$

(15) $$\mathrm{TPR}=\frac{\mathrm{TP}}{\mathrm{TP}+\mathrm{FN}}$$

(16) $$\mathrm{FPR}=\frac{\mathrm{FP}}{\mathrm{TN}+\mathrm{FP}}$$

### 4.3. Experimental Data

In this study, real datasets and artificially synthesized datasets were used to validate the superiority and reliability of the proposed method. The real datasets were obtained from the publicly available datasets in the UCI and KEEL machine learning repositories. Specifically, four datasets (Musk, Thyroid, Pendigits, and Pima) from the UCI database and eight datasets (Movement, Coil2000, Optdigits, Spambase, Spectfheart, Texture, Satimage, Ring, and Twonorm) from the KEEL database were utilized. The specific datasets used are listed in Table 2, including the dataset names, sample sizes (proportion of outliers), number of attribute features, and the number of classes. In this experiment, for datasets with two classes, the class with a larger number of samples was considered the normal class. For datasets with multiple classes, the classes with the largest and smallest number of samples were designated as the normal class and the outlier class, respectively. In the case of the Ring and Twonorm datasets, the class with the smallest number of samples accounted for more than 40% of the total samples, and a random selection of 10% of those samples was chosen as the outlier class.

This study also employed six manually generated datasets, labeled as A, B, C, D, E, and F, as shown in Table 3. Each dataset consists of 5000 objects, with an anomaly ratio of 0.1. The dimensions of each dataset were 5, 10, 20, 40, 80, and 160, respectively. During the data generation process, the normal points in each dataset were formed by 2 to 5 groups or clusters, and this was achieved by following their respective distributions to avoid an overfitting of the detection results.

### 4.4. Experimental Contents

The LOF, SOD, mRMRD-LOF, SFB, and EOEH algorithms were individually applied to conduct the experiments on the 12 publicly available real datasets from the UCI and KEEL machine learning repositories in order to validate the feasibility of the EOEH algorithm.

For the LOF and SOD algorithms, the parameter of k was set to integer values ranging from 5 to 50, and the best detection output was selected as the experimental result. For the mRMRD-LOF algorithm, the parameters recommended by the author were used, with p = 2, m = 5, and MinPts = 50. For the SFB algorithm, the choice of parameters depends on the dimensionality of the data. In high-dimensional datasets, a relatively large number of groups M was favored to avoid a loss of information. On the other hand, in relatively low-dimensional datasets, a smaller number of groups M was preferred to avoid redundant subspaces. Therefore, the M × T parameters for this algorithm were dynamically selected to achieve optimal detection results for the different datasets.

**Figure 3:**
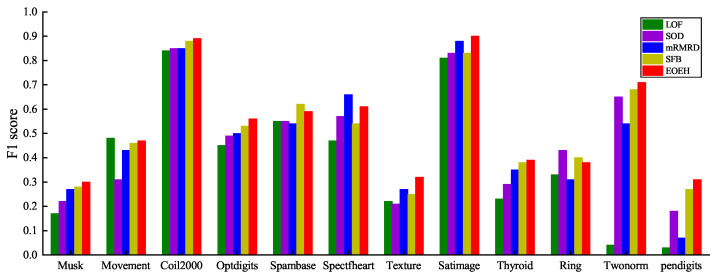
The F1 score of the five methods on the six datasets of Musk, Movement, Coil2000, Optdigits, Spambase, Spectfheart are visualized in the following graph.

In the experiments, the parameters of the EOEH algorithm were determined through multiple trials in the validation section. The number of sub-samples T was set to 25; the sample size in each sub-sample μ was set to 10% of the total number of samples |Y|; the abnormal information entropy weight α was set to 1.5; the normal information entropy weight β was set to 1; and the neighborhood parameter K was set to 50. The detection results are shown in Figure 3.

By examining the results presented in Table 4, it is evident that the EOEH algorithm consistently outperforms other detection algorithms, as indicated by the bolded F1 score for each dataset (which represents the best performance result for each dataset). The EOEH algorithm achieves an overall better detection performance compared to the other algorithms, with EOEH consistently maintaining the highest F1 score among all algorithms for 8 of the 12 datasets. Additionally, for the remaining 4 datasets, the EOEH algorithm exhibits a similarly high F1 score, demonstrating its feasibility and effectiveness in anomaly detection, even in datasets where it is not the best performer. The algorithm’s improved handling of data sparsity in high-dimensional spaces further emphasizes its potential for continued research and development.

To better illustrate the excellent performance of the proposed algorithm in high-dimensional anomaly detection, this article further explores detection performance with particular attention to data dimensionality. In this study, experiments were conducted using artificially synthesized datasets to investigate the impact of increasing dimensionality values on the detection performance.

**Figure 4:**
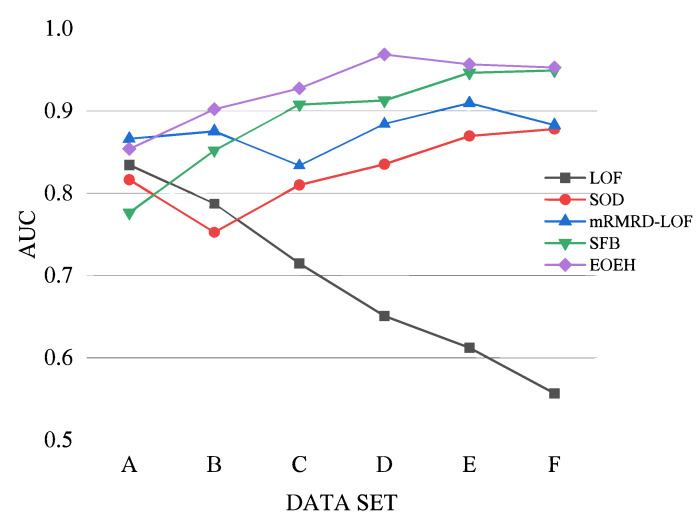
Visualization of AUC values for five algorithms on datasets of different dimensions.

From the results presented in Table 5 and Figure 4, it is clear that the EOEH algorithm has a slightly lower detection performance than the mRMRD-LOF algorithm on low-dimensional datasets. However, as dimensionality increases, the EOEH algorithm consistently outperforms other anomaly detection algorithms. The performance of LOF decreases as the dimensionality of the fixed-size dataset increases. This is because LOF detects outliers based on density in the entire feature space. As the number of irrelevant dimensions increases, the impact of the “curse of dimensionality” [9] becomes more prominent, thus hiding many of the points with anomalous behavior. The accuracy of SOD varies within a small range and does not exhibit a significant decreasing trend. However, throughout the experimental process, it became clear that the precision of the detection results was affected by the inclusion of some irrelevant dimensions in the subspaces selected by SOD. The mRMRD-LOF algorithm achieves a high detection performance in high-dimensional data; however, the algorithm only selects a single subspace to facilitate the discovery of outliers, which may reduce the robustness of the algorithm in certain cases. The SFB algorithm also performs well in detecting high-dimensional data, but its efficiency is lower due to the additional processing required during the generalization phase. With the increase in data dimension, the sparsity of high-dimensional space gradually has a negative impact on traditional detection algorithms. The detection effect of the SFB algorithm is poor when the data dimension is low, and there is a big gap with the EOEH algorithm model proposed in this paper. The reason is that SFB uses dynamic feature bagging. When there are few or no irrelevant dimensions in the data, SFB’s dynamic selection of subspace partitions and detectors is not always efficient. When the data dimension increases by leaps and bounds, SFB can dynamically select the most effective basic detector for each subspace.

Comparative experiments were conducted to investigate the variation in the runtime costs of the EOEH algorithm, LOF algorithm, and SFB algorithm with increasing dimensions. The experimental results are presented below.

**Figure 5:**
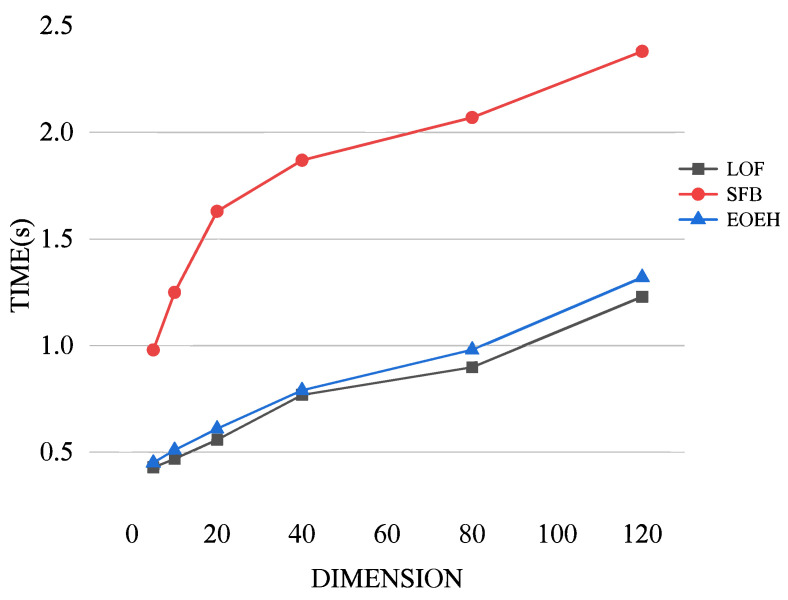
A visualization of the three algorithms’ run-time costs when utilized on datasets with different dimensions.

From Figure 5, it can be observed that, as data dimensionality increases, the runtime of all three algorithms gradually increases. Compared to the significantly higher runtime cost of the SFB algorithm, which is based on FB, the EOEH algorithm incurs only a slightly higher time overhead than the basic LOF algorithm while achieving a higher detection performance. This validates the superiority of the proposed algorithm.

The main process of this algorithm is divided into two parts: (1) abnormal feature subspace and feature weight vectors based on sub-sampling to generate data points; and (2) HPLOF outlier detection based on sub-sampling. In general, ensemble-based detection methods require more computation time than individual base detectors. However, the sub-sampling based ensemble proposed in this paper is faster and requires less resources than other types of ensembles. Among them, for a dataset with a data size of *n*, when calculating the abnormal feature subspace and feature weight vector of data points, the time complexity is $O\left(n^{2}\right)$ because the distance of all points needs to be calculated. However, this algorithm adopts the form based on sub-sampling. Since the distance calculation is transferred from the total sample set to the sub-sample set, the total time cost is sometimes even less than the time cost of running on the original dataset. If so, then the time complexity of process (1) is $\left(n^{2}\cdot p\cdot T\right)\left(p=\mu /n,0<p<1,1<T\ll n\right)$, where μ represents the number of samples in each sub-sample set, p represents the ratio of the number of samples in the sub-sample set to the total number of samples, *T* represents the number of sub-sample sets, and n represents the total number of samples. For process (2), the HPLOF algorithm has the same time complexity as LOF $O\left(n^{2}\right)$. Therefore, its time complexity is also $\left(n^{2}\cdot p\cdot T\right)\left(p=\mu /n,0<p<1,1<T\ll n\right)$. For example, in this experiment, the algorithm used only 10% of the original dataset in each subsample set, for a total of 25 subsample sets. Therefore, the integration time is about 2.5 times that of the LOF algorithm. In summary, the time complexity of the EOEH algorithm is $\left(n^{2}\cdot p\cdot T\right)\left(p=\mu /n,0<p<1,1<T\ll n\right)$, which can be approximately equal to $O\left(n^{2}\right)$.

The impact of the neighborhood parameter K on the detection performance of the proposed EOEH method was analyzed by conducting experiments on real datasets (including Musk, Texture, and Pendigits), and thus the changes in detection performance with varying values of the main parameter K in the algorithm can be analyzed. In these experiments, the number of sub-samples T was set to 25, with each sub-sample set containing 10% of the total samples |Y|, and the weights for abnormal information entropy α and normal information entropy β were set to 1.5 and 1, respectively.

**Figure 6:**
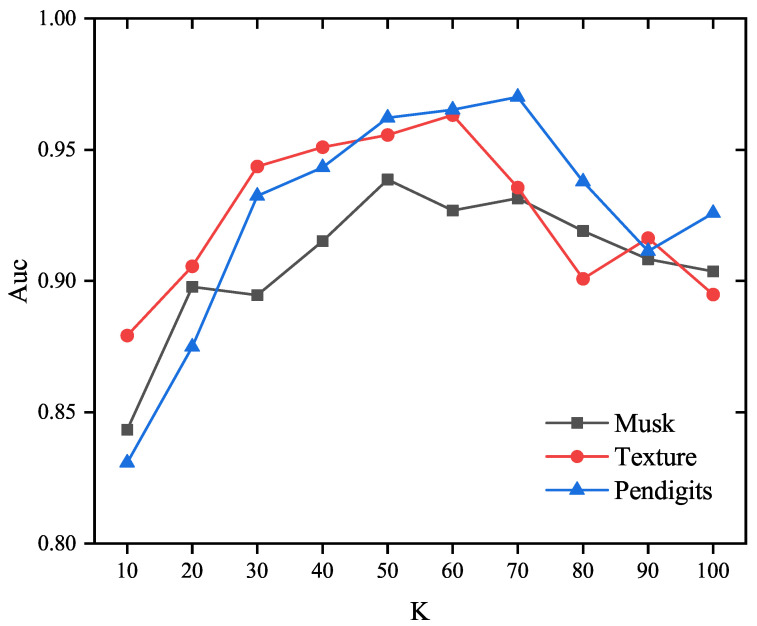
The influence of parameter K on the accuracy of the proposed method (T = 25, μ=10%|Y|,α=1.5,β=1).

From Figure 6, it can be observed that, in the three datasets, as the parameter K increases from 10 to around 60, the detection performance of the EOEH algorithm gradually improves. However, when K exceeds 70, the detection performance begins to decline. Excessively high values for the neighborhood parameter only lead to increased computational costs for the algorithm. Through multiple experiments, it will be possible to identify the optimal value of K that achieves the best EOEH algorithm performance in terms of both detection performance and execution time.

## 5. Conclusions

This paper proposes a new anomaly detection algorithm model called EOEH. The algorithm first performs random secondary subsampling on the available data, and then utilizes information entropy to calculate the dimensions’ space weights. It generates corresponding anomaly feature subspaces for each sub-sample set, and it calculates the weighted distances based on the feature subspaces of the data objects. Using the weighted distances, a newly designed high-precision local outlier factor (HPLOF) anomaly detector was employed. Finally, the HPLOF values of data objects on different sub-samples were integrated to obtain the anomaly scores. Through experiments conducted on both real datasets and synthetic datasets, the proposed algorithm demonstrates significant improvements in detection performance and runtime compared to other state-of-the-art anomaly detection algorithms.

However, the most significant challenge faced by this algorithm is the issue of determining optimal parameters. To address this, it is important to develop an effective and adaptive method to automatically determine the optimal parameters, which could further enhance the performance of the EOEH algorithm.

## Figures and Tables

**Table 1:** A confusion matrix is used to describe the classification results of a classification problem.

Real Class	Prediction Class	Index
1	1	True Positive (TP)
0	1	False Positive (FP)
1	0	False Negative (FN)
0	0	True Negative (TN)

**Table 2:** A variety of real-world datasets in the UCI and KEEL machine learning libraries.

Data Set	Data Size (Anomaly %)	Dimension	Classes
Musk	3062 (0.03)	166	5
Movement	360 (0.06)	90	15
Coil2000	9389 (0.05)	85	2
Optdigits	1014 (0.28)	64	10
Spambase	4597 (0.01)	57	2
Spectfheart	267 (0.21)	44	2
Texture	4755 (0.009)	40	7
Satimage	4577 (0.008)	36	6
Thyroid	7036 (0.01)	21	3
Ring	4564 (0.06)	20	2
Twonorm	4144 (0.08)	20	2
Pendigits	6870 (0.02)	16	10

**Table 3:** Synthetic datasets with different dimensions.

Data Set	Data Size (Anomaly %)	Dimension
A	5000 (0.1)	5
B	5000 (0.1)	10
C	5000 (0.1)	20
D	5000 (0.1)	40
E	5000 (0.1)	80
F	5000 (0.1)	160

**Table 4:** The F1 score of the five algorithms (LOF, SOD, mRMRD-LOF, SFB, and EOEH) on 12 real-world datasets.

Data Set	LOF	SOD	mRMRD	SFB	EOEH
Musk	0.17	0.22	0.27	0.28	0.30
Movement	0.48	0.31	0.43	0.46	0.47
Coil2000	0.84	0.85	0.85	0.88	0.89
Optdigits	0.45	0.49	0.50	0.53	0.56
Spambase	0.55	0.55	0.54	0.62	0.59
Spectfheart	0.47	0.57	0.66	0.54	0.61
Texture	0.22	0.21	0.27	0.25	0.32
Satimage	0.81	0.83	0.88	0.83	0.90
Thyroid	0.23	0.29	0.35	0.38	0.39
Ring	0.33	0.43	0.31	0.40	0.38
Twonorm	0.04	0.65	0.54	0.68	0.71
Pendigits	0.03	0.18	0.07	0.27	0.31

**Table 5:** Table of results of AUC values for five algorithms on datasets of different dimensions.

Data Set	LOF	SOD	mRMRD	SFB	EOEH
A	0.8344	0.8165	0.8661	0.7764	0.8541
B	0.7875	0.7528	0.8754	0.8517	0.8517
C	0.7148	0.8101	0.8338	0.9077	0.9273
D	0.6509	0.8353	0.8844	0.9129	0.9684
E	0.6123	0.8697	0.9094	0.9465	0.9566
F	0.5569	0.8782	0.8827	0.9493	0.9527

## Data Availability

The data presented in this study are available on request from the corresponding authors.
